# Structural evidence that RNA contributes to polymorphism of tau amyloid fibrils

**DOI:** 10.1016/j.isci.2026.115501

**Published:** 2026-03-26

**Authors:** Romany Abskharon, Yi Xiao Jiang, Michael R. Sawaya, Peng Ge, Jeffrey Zhang, David R. Boyer, Joshua L. Dolinsky, Justin Pi, Duilio Cascio, Feng Guo, David S. Eisenberg

**Affiliations:** 1 Department of Chemistry and Biochemistry, Department of Biological Chemistry, UCLA-DOE Institute, Molecular Biology Institute, UCLA, Los Angeles, CA 90095, USA; 2 Department of Biological Chemistry, Molecular Biology Institute, UCLA, Los Angeles, CA 90095, USA

**Keywords:** biochemistry, structural biology, experimental systems for structural biology

## Abstract

RNA colocalizes with tau deposits in Alzheimer’s disease (AD) and drives tau aggregation *in vitro*. Previously, we determined a cryogenic-electron microscopy (cryo-EM) structure of fibrils of full-length tau bound to unfractionated mammalian RNA, revealing a small tau C-terminal core. Here, we present the cryo-EM structure of fibrils of full-length recombinant tau bound to unfractionated mammalian RNA seeded by AD-extracted tau fibrils. This structure reveals an expanded tau C-terminal core resembling AD tau fibrils. RNA sequencing identified 18S ribosomal RNA as the primary fibril-bound species. Next, we determined the cryo-EM structure of fibrils of full-length recombinant tau bound to mammalian 18S ribosomal RNA, revealing a core that consists of the R2 to R4 repeat domains previously seen in pathological tau fibrils. All our recombinant RNA-tau fibrils dissolve upon RNase treatment. Tau fibrils adopt distinct folds in the presence of different RNAs, suggesting RNA is a cofactor capable of shaping tau fibril polymorphism.

## Highlights

• Cryo-EM reveals structures of full-length tau fibrils in complex with mammalian RNA
• RNA acts as a molecular glue to stabilize RNA-tau fibrils
• RNA modulates the structure of tau amyloid fibrils

## Graphical abstract

**Figure:**
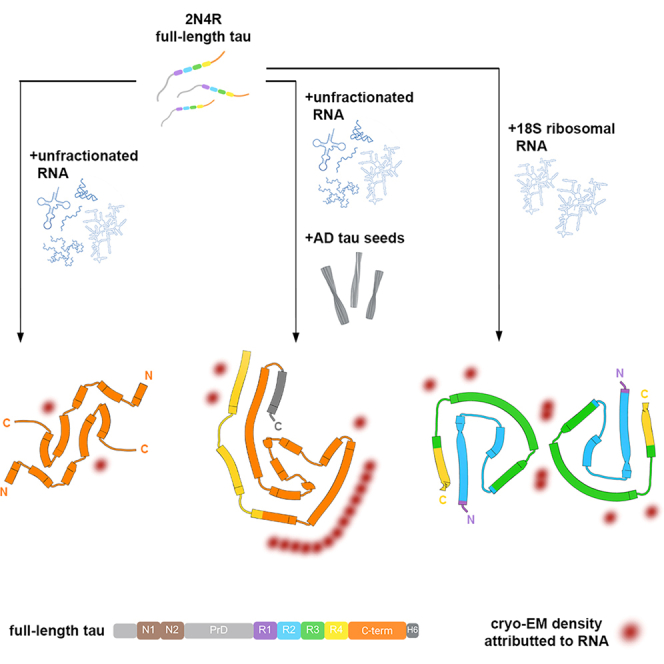

## Introduction

Alzheimer’s disease (AD) is a progressive neurodegenerative disorder presenting with memory loss and pathologically defined by plaques containing amyloid-β and neurofibrillary tangles containing tau amyloid fibrils.^1^^,^^2^^,^^3^^,^^4^ Tau has a physiological function of microtubule stabilization;^5^ however, unknown factors cause its pathological aggregation in AD, tauopathies, and other neurodegenerative diseases.^6^ One of the cofactors of tau aggregation is RNA, associated with tau lesions in AD and Pick’s disease,^7^^,^^8^ as well as in cell models of tau aggregation.^9^^,^^10^ RNA induces *in vitro* tau fibril formation,^11^^,^^12^^,^^13^^,^^14^ serving as an anionic complement to lysine-rich, cationic tau, lowering the electrostatic barrier to tau aggregation. RNA also drives intrinsically disordered tau into liquid-liquid phase separation in cells.^15^^,^^16^ Dense liquid droplets with high local protein concentration are optimal environments for aggregation and may serve as an intermediate toward tau fibril formation.^17^^,^^18^ One study found that the seeding capacity of AD tau is sensitive to RNase, suggesting a role for RNA in the formation and maintenance of tau fibrils.^19^ We recapitulated this observation (Figure S1), underscoring the need to better understand pathological RNA-tau interactions at a molecular level. Here, we complexed full-length tau with RNA species to form amyloid fibrils and determined their structures using cryogenic-electron microscopy (cryo-EM). These structures illustrate how RNA promotes tau aggregation.

## Results

### Full-length tau incubated with unfractionated mouse liver RNA and seeded by AD tau forms fibrils sensitive to RNase

**Figure 1:**
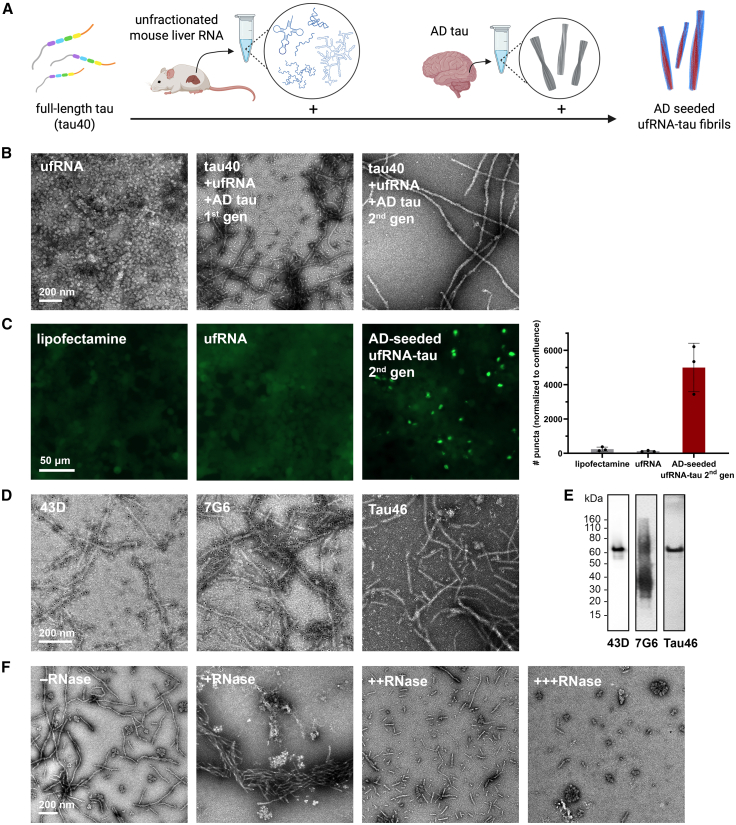
Full-length tau incubated with unfractionated mouse liver RNA and seeded by AD tau forms fibrils sensitive to RNase (A) Schematic of full-length 2N4R tau (tau40) fibril formation with unfractionated mouse liver RNA and AD tau seeds (AD-seeded ufRNA-tau fibril). (B) Negative stain TEM micrographs of unfractionated RNA (ufRNA) alone, 1st and 2nd generation AD-seeded ufRNA-tau fibrils. Scale bars, 200 nm. (C) Confocal images and quantification of tau biosensor cell seeding by AD-seeded ufRNA-tau fibrils. Scale bars, 50 μm. Data are represented as mean ± SD. (D) Immunogold labeling of AD-seeded ufRNA-tau fibrils using 43D (epitope: tau 1–100), 7G6 (epitope: tau 299–303 and 362–366, HVPGG sequence of microtubule binding regions R2 and R4), and Tau46 (epitope: tau 404–441, C-terminal domain) antibodies. Scale bars, 200 nm. (E) Immunoblotting of denatured AD-seeded ufRNA-tau fibrils using 43D, 7G6, and Tau46 antibodies. (F) Negative stain TEM micrographs of AD-seeded ufRNA-tau fibrils treated with 1:0 (–RNase), 1:0.1 (+RNase), 1:1 (++RNase), and 1:10 (+++RNase) molar ratios of fibril:RNase A at 37 °C for 2 h. Scale bars, 200 nm.

We previously determined the cryo-EM structure of fibrils of full-length tau formed with unfractionated (uf) mouse liver RNA (unseeded ufRNA-tau fibrils), revealing a small C-terminal core which we speculated may nucleate pathogenic tau conformations.^14^ Here, to test whether the addition of tau fibrils extracted from the postmortem brain of an AD patient (AD tau) could template the formation of the AD fold, we mixed full-length 2N4R tau (tau40) with uf mouse liver RNA and AD tau seeds to incubate at 37 °C with constant shaking for 2 days (AD-seeded ufRNA-tau fibrils, Figure 1A). We termed these first-generation fibrils, which were clumped and unsuitable for cryo-EM. We used these fibrils to seed a second generation using tau40, uf mouse liver RNA, and AD tau seeds, as described for the first generation. Second generation produced cleaner, single protofilament fibrils (Figure 1B) that seeded tau biosensor cells^20^ (Figure 1C). AD-seeded ufRNA-tau fibrils were immunogold labeled by antibodies 43D (epitope: tau 1–100) and 7G6^21^ (epitope: tau 299–303 and 362–366, HVPGG sequence of microtubule binding domains R2 and R4) but not labeled by Tau46 (epitope: tau 404–441, C-terminal domain), suggesting that the C-terminal domain forms the amyloid core and is not accessible (Figure 1D). Under denaturing conditions, fibril samples were detected by all three antibodies, confirming they are composed of full-length tau (Figure 1E). Like unseeded ufRNA-tau fibrils,^14^ these fibrils disassemble upon incubation with the increasing concentration of RNase at 37 °C for 2 h (Figure 1F). We conclude that AD-seeded ufRNA-tau fibrils are reversible amyloids in which RNA serves as molecular glue.

### AD-seeded ufRNA-tau fibrils reveal RNA interfaced with a C-terminal core

**Figure 2:**
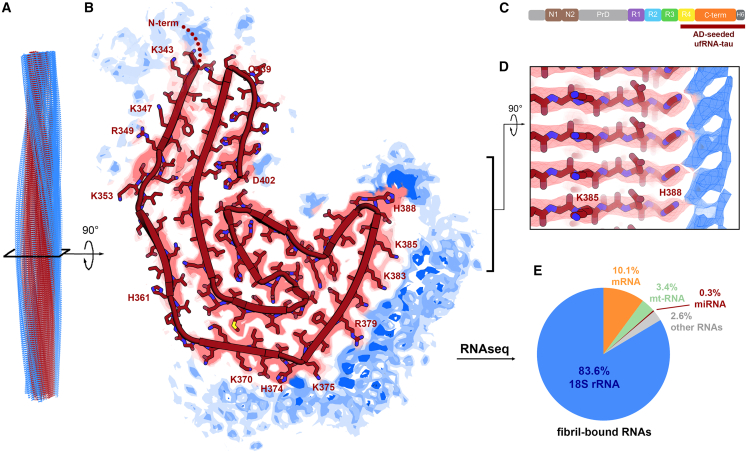
Cryo-EM structure of full-length tau fibril in complex with unfractionated RNA and seeded by AD tau (A) Reconstructed side view of the single protofilament (red) and residual densities (blue). (B) Sharpened cryo-EM map and refined atomic model of AD-seeded ufRNA-tau fibril. Residual densities (blue) suggest fibril-bound RNA. (C) Fibril core consists of R4 and C-terminal domain residues of tau and the C-terminal His-tag. (D) Side view of layers of H388 adjacent to prominent residual density (blue). (E) RNA sequencing of fibril-bound RNA species.

We determined a 3.1 Å cryo-EM structure of AD-seeded ufRNA-tau fibrils. This single protofilament fibril has an ordered core that consists of R4 and C-terminal domain residues of tau and the C-terminal his-tag, from S341 to H449 (Figures 2, S2A, S2B, S3A, and S4A; Table 1). This core completely encompasses the span of our previously reported unseeded ufRNA-tau fibrils (PDB 7sp1),^14^ which is also centered near the C-terminus (residues 391–426). We noticed a structural motif is conserved: Residues E391 to S400 form a consecutive pair of β strands joined by a 90° kink between Y394 and K395. When superimposed, these segments have an RMSD of 1.14 Å (Figure S5A).

**Table 1:** Cryo-EM data collection, processing, and validation statistics

	AD-seeded ufRNA-tau fibril (PDB 9o8e)	Unseeded 18S rRNA-tau fibril (PDB 9o8h)
**Data collection and processing**		

*Magnification*	×64,000	×81,000
*Voltage (kV)*	300	300
*Electron exposure (e*^*–*^*Å*^*−2*^*)*	36	60
*Defocus range (μm)*	0.60–2.3	0.60–3.5
*Pixel size (Å)*	1.077	1.07
*Symmetry imposed*	C1; Helical	C1; Helical
*Helical rise (Å)*	4.839	2.39525
*Helical twist (°)*	−1.03	179.634
*Initial particle images (no.)*	280,270	545,242
*Final particle images (no.)*	54,683	25,873
*Map resolution (Å)*	3.1	3.3
*FSC threshold*	0.143	0.143
*Map resolution range (Å)*	200–3.1	200–3.3

**Refinement**		

*Initial model used*	*De novo*	*De novo*
*Model resolution (Å)*	3.1	3.3
*FSC threshold*	0.5	0.5
*Model resolution range (Å)*	200–3.2	200–3.4
*Map sharpening B factor (Å*^*2*^*)*	−98	−65

**Model composition**		

*Non-hydrogen atoms*	4165	6380
*Protein residues*	545	850
*RNA residues*	0	0

**B factors (Å2)**		

*Protein*	98.1	103.2

**R.m.s. deviations**		

*Bond lengths (Å)*	0.003	0.013
*Bond angles (°)*	0.64	0.98

**Validation**		

*MolProbity score*	2.11	2.73
*Clashscore*	13.6	12.5
*Poor rotamers (%)*	0	5.5

**Ramachandran plot**		

*Favored (%)*	92.5	89.7
*Allowed (%)*	7.5	10.3
*Disallowed (%)*	0	0

We noted a gross resemblance between the C-shapes of AD-seeded ufRNA-tau fibrils and the paired helical filament (PHF, PDB 5o3l)^22^ of AD tau seeds. The AD PHF core spans a shorter, more N-terminal residue range (V306-F378); nevertheless, 25-residue-long C-shaped segments from the two fibrils (AD-seeded ufRNA-tau fibril N359-K383 and AD PHF V318-E342) superimpose with an RMSD of 1.71 Å (Figure S5B). Also, we noted similar dimensions of the long steric zipper that mates N- and C-terminal β-strands in both fibrils (Figure S5C). These similarities suggest that AD tau imparted segments of its structure onto different sequences in the AD-seeded ufRNA-tau fibril progeny. We offer a speculative model for how C-terminal residues of tau nucleated a small core in the presence of uf RNA, then AD tau influenced its expansion into a larger C-shaped fold (Figure S5D).

The cryo-EM map of AD-seeded ufRNA-tau fibrils contains evidence of RNA binding to tau. Tubes of residual density run parallel to the fibril axis, most prominently at the surface of the tau fibril that exhibits the most positive charge (Figures 2B and S4A). We attribute this residual density to the negatively charged phosphate backbone of RNA in complex with the positively charged lysine, arginine, and histidine residues of tau (K370, H374, K375, R379, K383, K385, H388), which project into it. RNA is not well resolved because the spacing between nucleotides is incommensurate with the helical averaging 4.8 Å spacing between tau molecules defined by the backbone hydrogen bonds (Figure 2D). Seeking biochemical evidence that RNA is in complex with tau fibrils, we pelleted and washed ufRNA-tau fibrils, then detected fibril-bound RNA by spectroscopy (Figure S6A).

### C-terminal core of AD-seeded ufRNA-tau fibrils binds primarily 18S ribosomal RNA

To identify RNA species in complex, we isolated AD-seeded ufRNA-tau fibrils by centrifugation, washed away unbound RNA, and sequenced fibril-bound RNA (see STAR Methods). Strikingly, 18S ribosomal RNA (rRNA) is the major fibril-bound species (Figure 2E). In addition, protein-coding mRNAs, mitochondrial RNAs, and miRNAs were identified.

### Full-length tau incubated with mouse liver 18S rRNA forms fibrils recognized by AD-specific GT-38 antibody

**Figure 3:**
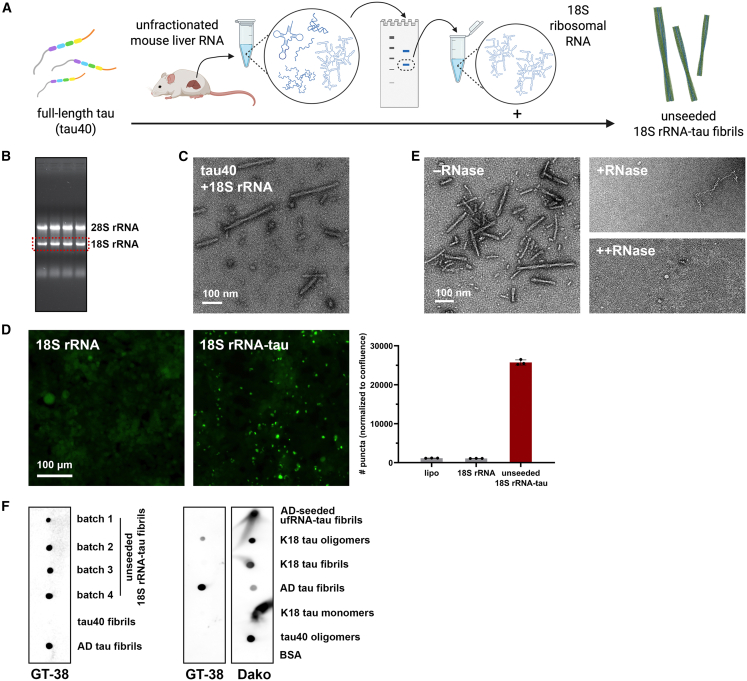
Full-length tau incubated with mouse liver 18S rRNA forms fibrils recognized by GT-38 antibody (A) Schematic of full-length 2N4R tau (tau40) fibril formation with mouse liver 18S ribosomal RNA (unseeded 18S rRNA-tau fibril). (B) Agarose gel electrophoresis of isolated unfractionated mouse liver RNA on agarose gel. 18S rRNA bands (red rectangle) were extracted and purified for tau fibril formation. (C) Negative stain TEM micrograph of unseeded 18S rRNA-tau fibrils. Scale bars, 100 nm. (D) Confocal images and quantification of tau biosensor cell seeding by unseeded 18S rRNA-tau fibrils. Scale bars, 100 μm. Data are represented as mean ± SD. (E) Negative stain TEM micrographs of 18S rRNA-tau fibrils treated with 1:0.1 (+RNase) and 1:1 (++RNase) molar ratios of fibril:RNase A at 37 °C for 2 h. Scale bars, 100 nm. (F) Dot blot using GT-38 antibody recognized unseeded 18S rRNA-tau fibrils (left), but not other recombinant tau samples of various conformations (right). Dako (epitope: tau 243–441) blots for total tau.

From our work with AD-seeded ufRNA-tau fibrils, we were motivated to study interactions of tau with 18S rRNA alone, without the influence of AD tau seeds. We isolated 18S rRNA from uf mouse liver RNA and showed its direct binding to tau40 monomers by gel shift, while other RNA species did not (Figures S6B and S6C). We mixed tau40 and 18S rRNA to incubate at 37 °C with constant shaking for 3 days (unseeded 18S rRNA-tau fibrils, Figures 3A and 3B). 18S rRNA and tau formed double protofilament fibrils that seeded tau biosensor cells and dissolved in the presence of increasing concentration of RNase at 37 °C for 2 h (Figures 3C–3E). Interestingly, we observed strong staining of 18S rRNA-tau fibrils in an immunoblot with GT-38, an antibody specific for AD and chronic traumatic encephalopathy (CTE) tau pathology but not FTLD-tau pathology from corticobasal degeneration, progressive supranuclear palsy, or Pick’s disease^23^^,^^24^ (Figure 3F). Other recombinant tau monomer, oligomer, and fibril samples were not recognized by GT-38, including the AD-seeded ufRNA-tau fibrils.

### Unseeded 18S rRNA-tau fibrils are paired helical filaments with a core of primarily R2 and R3 domains

We determined a 3.3 Å cryo-EM structure of unseeded 18S rRNA-tau fibrils. The double protofilaments are related by pseudo-2_1_ symmetry, and the ordered core consists of R1 to R4 domain residues of tau, G273 to S341 (Figures 5, S2C, S2D, S3B, and S4B; Table 1). These fibrils are paired helical filaments; however, they are distinct from AD PHFs both in conformation and residue composition. uf RNA drove the formation of C-terminal cores, 18S rRNA alone yielded a core of primarily R2 and R3 domains. In fact, there is an overlap of only two residues, S341 and E342, between the cores of unseeded 18S rRNA-tau and AD-seeded ufRNA-tau fibrils. The cryo-EM map at the C-terminus of the ordered core revealed alternative conformations for residues N327 to E342 (Figure S7), indicating flexibility in this connector to the fuzzy coat. We noticed the R2 domain PGGG turn motif of residues I297 to V313 had been observed in other tau amyloid structures: the GGT-PSP-Tau fold (GPT fold, PDB 7p6a)^25^ and a dGAE fibril formed *in vitro* with pyrophosphate (PBD 7ql2)^26^ (Figure S8). A disulfide bond was formed between C291 and C322 due to the lack of a reducing agent during fibril formation. Under reducing conditions with freed cysteines, 18S rRNA-tau fibrils could resemble the GPT or another tauopathy fold even more closely.

Known to distinguish AD and CTE pathology (which share similar tau folds) from pathology of other tauopathies (in which tau folds differ),^23^^,^^24^^,^^25^ GT-38 antibody recognizes unseeded 18S rRNA-tau fibrils. Our cryo-EM structure provides an opportunity to examine potential epitopes of GT-38, which could inform its use at the molecular, neuropathological, and clinical levels. The AD PHF and unseeded 18S rRNA-tau fibril both have C-shaped folds with the outer β-strand layers composed of R3 and R4 domain residues. Three segments, or parts of these segments, could serve as potential GT-38 binding sites: Q307 to K311, K317 to G323, and N327 to E342 (Figure S9).

**Figure 4:**
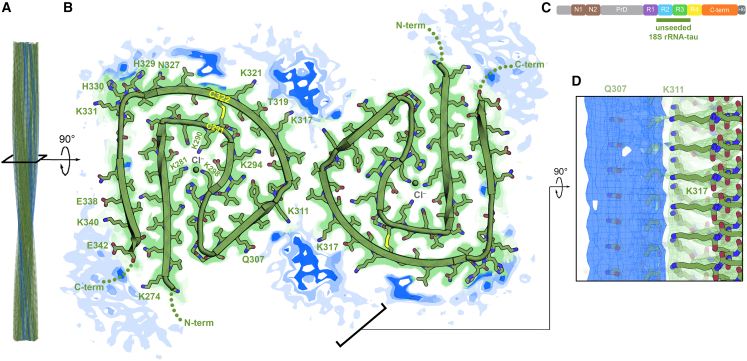
Cryo-EM structure of full-length tau fibril in complex with 18S rRNA (A) Reconstructed side view of the double protofilament (green) and residual densities (blue). (B) Sharpened cryo-EM map and refined atomic model of unseeded 18S rRNA-tau fibril. Residual densities (blue) suggest fibril-bound RNA. (C) Fibril core consists mainly of R2 to R3 of tau. (D) Side view of layers of K317 (foreground) and K311 (background) adjacent to prominent residual density (blue).

The cryo-EM map of unseeded 18S rRNA-tau fibrils also presents evidence of RNA’s phosphate backbone interacting with tau’s charged residues. The most prominent residual density is beside the dimer interface of the protofilaments (Figures 4B and S4B), contacted by R3 domain lysine residues K311, K317, and K321. K317 and K321 have been observed to contact similar residual densities in AD, progressive supranuclear palsy (PSP, PDB 7p65)^25^ and argyrophilic grain disease (AGD, PDB 7p6d)^25^ tau fibrils (Figure S10). Notable residual densities are found near R3 domain histidine residues H329 and H330, which may be additional RNA binding. As before, RNA is poorly resolved and appears as tubes of residual density along the fibril axis (Figure 4D). Surprisingly, R2 domain lysine residues K281, K290, and K298 are buried together, their charges are offset by two aspartates and an apparent chloride ion.

### Computation suggests RNA stabilizes RNA-tau fibrils by compensating charged residues

**Figure 5:**
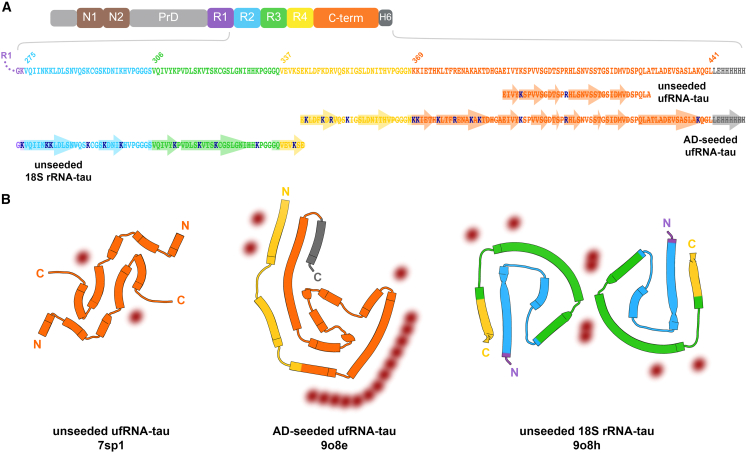
Amyloid cores of RNA-tau fibrils (A) Full-length 2N4R tau protein (tau40) domains and amino acid sequences for microtubule binding repeats R1 to R4, C-terminal domain, and his-tag (H6). Amyloid core sequences of unseeded ufRNA-tau fibril (PDB 7sp1), AD-seeded ufRNA-tau fibril, and unseeded 18S rRNA-tau fibril are aligned, with β strands annotated as arrows. Lysine and arginine residues are highlighted (dark blue). (B) Cartoons of amyloid cores of unseeded ufRNA-tau fibril (left), AD-seeded ufRNA-tau fibril (middle), and unseeded 18S rRNA-tau fibril (right). Residual densities (red) indicate potential RNA interactions.

**Figure 6:**
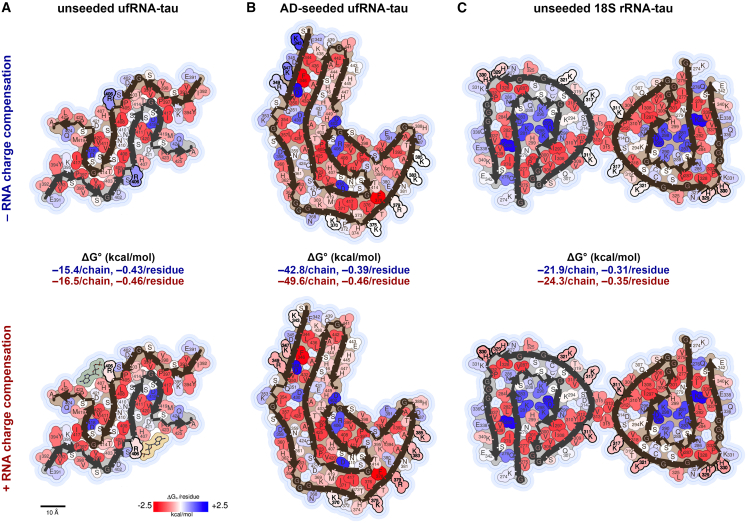
RNA stabilizes RNA-tau fibrils by compensating for charged residues Solvation energy maps and calculated energies of (A) unseeded ufRNA-tau fibril, (B) AD-seeded ufRNA-tau fibril, and (C) unseeded 18S rRNA-tau fibril before (top) and after (bottom) accounting for charge compensation of lysines, arginines, and histidines by RNA. Charge compensation was attributed to unseeded ufRNA-tau fibril residue R406, AD-seeded ufRNA-tau fibril residues K343, K347, R349, K370, K375, R379, K783, and K385, and unseeded 18S rRNA-tau fibril residues K311, K317, K321, H329, and H330 (bolded).

Our biochemical experiments demonstrate that RNA is an integral component of RNA-tau fibrils (Figures 1F and 3E), and our cryo-EM structures confirm RNA in complex along the sides of tau fibrils (Figure 5; Table 2). Furthermore, we can approximately quantify RNA stabilization via solvation energy calculations. To approximate the effect of RNA compensating positive charges, we nullified unfavorable atomic solvation parameters for lysines, arginines, and histidines for which we have evidence of RNA interaction based on the cryo-EM residual density (Figure 6). As expected, RNA charge compensation increases the overall stability (ΔG° per chain) and energetic efficiency (ΔG° per residue) of RNA-tau fibrils. More decisively, RNA stabilization of AD-seeded ufRNA-tau fibrils lowers ΔG° per chain from −42.8 kcal/mol to −49.6 kcal/mol, and lowers ΔG° per residue from −0.39 kcal/mol to −0.46 kcal/mol. Apparently, RNA charge compensation enables energetically favorable tau folds to exist, which may give rise to intermediates on the pathway to more stable fibrils.

**Table 2:** Comparison of RNA-tau fibrils

*Feature*	Unseeded ufRNA-tau fibril (PDB 7sp1, Abskharon et al., 2022)	AD-seeded ufRNA-tau fibril (PDB 9o8e)	Unseeded 18S rRNA-tau fibril (PDB 9o8h)
*Tau*	Tau40 with his-tag	Tau40 with his-tag	Tau40 with his-tag
*RNA*	mouse liver ufRNA	mouse liver ufRNA	mouse liver 18S rRNA
*Seed*	no seed	AD patient-extracted tau fibrils	no seed
*Tau fibril core residues*	391–426 (small C-term)	341–441 (expanded C-term)	273–341 (primarily R2 and R3)
*Protofilaments*	paired	Single	paired
*Tau fibril features*	unique S-shaped fold	C-shaped fold, different residues than AD PHF	C-shaped fold, different residues than AD PHF
*Positively charged tau residues in proximity to residual density*	R406, H407	K370, H374, K375, R379, K383, K385, H388	K311, K317, K321, H329, H330
*Interpretation*	*de novo* fibril formation with bulk RNA cofactor	AD fibril-templated fibril formation with bulk RNA cofactor	*de novo* fibril formation with 18S rRNA cofactor

### Computation suggests RNA-tau fibrils are less stable compared to *ex vivo* tau folds

**Figure 7:**
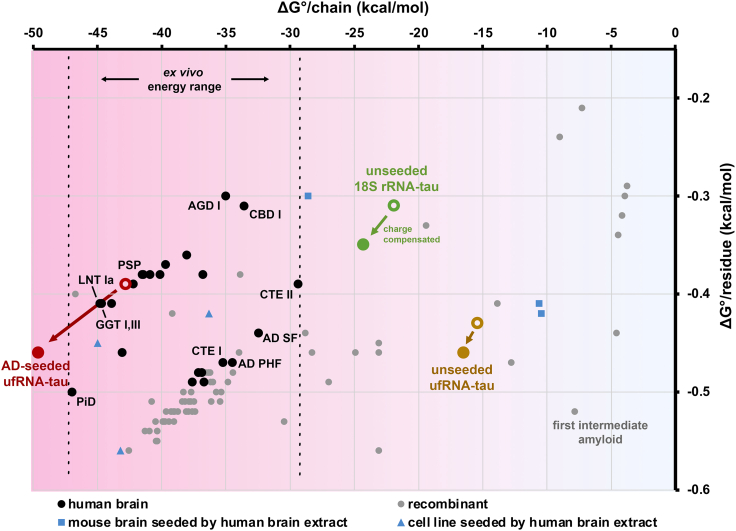
Tau fibril energy map shows that unseeded RNA-tau fibrils are less stable compared to *ex vivo* tau folds Tau fibril structures plotted in two dimensions based on solvation energy (ΔG°) per chain and per residue. Each point represents a tau fibril structure from human brain (black circles), recombinant protein (gray circles), mouse brain seeded by human brain extract (blue squares), or cell line seeded by human brain extract (blue triangles). RNA-tau fibrils are plotted for before (white fill) and after accounting for charge compensation by RNA (colored fill). ΔG° per chain of *ex vivo* tau fibrils ranges between approximately −29 to −47 kcal/mol (dotted lines). Solvation energies were tabulated from Amyloid Atlas (https://people.mbi.ucla.edu/sawaya/amyloidatlas/) on March 9, 2025, and recorded in Table S1.

To contextualize the solvation energy of RNA-tau fibrils, we tabulated from Amyloid Atlas (https://people.mbi.ucla.edu/sawaya/amyloidatlas/) and plotted all *ex vivo*, recombinant, mouse or cell line-derived tau fibril structures on two axes, ΔG° per chain and ΔG° per residue (Figure 7; Table S1). We plotted RNA-tau fibril energies before and after accounting for charge compensation by RNA, showing the effects of RNA stabilization. Notably, ΔG° per chain of *ex vivo* tau folds ranges between approximately −29 to −47 kcal/mol, more stable than the range of recombinant tau fibrils, which is approximately −3 to −47 kcal/mol. The first intermediate amyloid (FIA), a precursor of the AD and CTE folds,^27^ exhibits high stability on a per-residue basis, but it has a small core and thus weak overall stability with ΔG° per chain of −7.85 kcal/mol. The unseeded ufRNA-tau and 18S rRNA-tau fibril energies after RNA charge compensation lie between the FIA energy and range of stable *ex vivo* fibrils, with ΔG° per chain of −16.5 and −24.3 kcal/mol, respectively. Our tau fibril energy map indicates that RNA-tau fibril complexes fall within a range consistent with intermediate states prior to *ex vivo*, disease-associated folds. AD-seeded ufRNA-tau fibrils are very stable, with ΔG° per chain of −49.6 kcal/mol, presumably because it has been seeded by *ex vivo* fibrils.

## Discussion

RNA is a well-established cofactor of tau aggregation; however, little is known about the atomic structural basis of interactions between RNA and tau. To address this deficit, we formed full-length tau fibrils in complex with uf or 18S rRNA purified from mouse liver and determined their structures using cryo-EM. RNA-tau fibrils disassemble upon RNase treatment in a concentration-dependent manner, demonstrating that RNA is an integral component of these complexes. In all three of our recombinant RNA-tau fibril structures, we observed residual density in proximity to positively charged tau residues, mostly lysines, from the R2 to R4 and C-terminal domains (Figure 5). Some of these interactions are analogous to those between *ex vivo* tau fibrils and unidentified cofactors (Figure S10), perhaps suggesting that RNA-tau binding motifs observed here may or may not be present in pathogenic structures. There is no previous direct evidence of RNA bound to *ex vivo* tau fibrils, and the nature of RNA’s association with tau aggregates has remained an open question. Here, we show biochemical and structural evidence of RNA bound directly to tau fibrils in three *in vitro* conditions. RNA stabilizes tau fibrils by the electrostatic compensation of stacked charges of lysines, arginines, and histidines along the fibril axis. Our energy map (Figure 7) reveals that unseeded RNA-tau fibrils are less stable than *ex vivo* tau folds, possibly representing transient conformations that precede more stable tau structures.

In three varied conditions, RNA-tau fibril folds and core residues are distinct (Figure 5; Table 2), showing that RNA influences tau polymorphism in ways we do not yet fully understand. Tau fibrils grown with uf RNA, unseeded and AD-seeded, share a small, conserved motif. Comparing unseeded ufRNA-tau and 18S rRNA-tau fibrils, in which the only variable is the type of RNA, the tau cores are composed of completely different sequences. These RNA-induced tau folds have not been observed before in the ∼70 recombinant tau fibrils reported so far, indicating RNA allows tau to explore fold space that is inaccessible in its absence. This suggests that RNA may not only be a passive anionic cofactor, but possibly an active molecular determinant of tau fibril structures. Our observation of RNA affecting tau polymorphism *in vitro* raises the possibility that RNA contributes to the diversity of tau folds in neurodegenerative diseases. That is, specific RNAs, potentially in combination with other cofactors, tau post-translational modifications, and cellular elements, may drive particular tau fibril folds.

In a mixture with uf RNA, tau is mostly associated with 18S rRNA (Figure 2E), which is intriguing due to existing literature linking rRNA and tau. Tau binds to ribosomal proteins and RNAs,^28^^,^^29^ and pathological tau may impair ribosomal function and decrease translation in AD^30^^,^^31^^,^^32^^,^^33^ and other tauopathies.^34^^,^^35^^,^^36^ Our study provides structural support for a pathological interaction between full-length tau and rRNA. Further, this promotes a hypothesis that tau aggregation could be concurrent with its translation, while in contact with rRNA. It remains to be answered whether rRNAs induce tau aggregation or whether tau aggregates impede ribosomal function. Unseeded 18S rRNA-tau fibrils resemble the AD fold only in gross C-shape, and not in detail. Nevertheless, in the structure induced by 18S rRNA yields an epitope recognized by GT-38, an antibody specific for AD and CTE pathology.^23^^,^^24^ GT-38 may bind along the side of tau fibril cores, on solvent-accessible R3 and R4 domain residues (Figure S9).

In summary, our biochemical and structural study of three recombinant RNA-tau fibrils reveals that RNA binds, stabilizes, and steers polymorphism of tau fibrils. These findings suggest a connection between tau aggregation and rRNA.

### Limitations of the study

In our study, we explored the role of RNA in producing tau polymorphs observed in AD and tauopathies. The polymorphs observed here are not exact matches to known patient-extracted fibrils, and we are mindful of factors that may have contributed to structures different from those formed in human brains. First, recombinant tau40 includes a C-terminal 6-residue His-tag, which may exert influence on fibril structure. Structures of amyloid fibrils are sensitive to changes in protein sequence as well as changes in cellular environment. While the his-tag is in the AD-seeded ufRNA-tau fibril core, there is no prominent residual density in its proximity, indicating contact with RNA (Figure 2B). Second, the RNA is not from human sources but isolated from mouse liver, which we selected for its low levels of tau compared to other organs such as the brain.^37^ Human and mouse RNA are moderately conserved, although there may be interactions specific to human RNA not reflected here. Importantly, we note that the 18S rRNA of human and mouse are 99% conserved.^38^ The tau40 and mouse liver RNA are the same as those used in our previous study,^14^ allowing for the evaluation of the effect of single variables (AD tau seed, 18S rRNA). Third, due to the technical limitation that first-generation AD-seeded ufRNA-tau fibrils were not amenable to cryo-EM, we cannot rule out that first-generation fibrils could be structurally different from second-generation fibrils. First-generation fibrils were added to a mixture of identical components (tau40, ufRNA, AD tau) as a pseudo-continuation of the reaction that yielded second-generation fibrils, which were amenable to cryo-EM. We believe these limitations do not detract from our demonstration of different specificities of tau to different RNA molecules, shown through the molecular structures of tau in these complexes.

## Resource availability

### Lead contact

Requests for further information and resources should be directed to and will be fulfilled by the lead contact, David S. Eisenberg (david@mbi.ucla.edu).

### Materials availability

Reagents generated in this study are available from the lead contact upon reasonable request.

### Data and code availability

• Cryo-EM micrographs for AD-seeded ufRNA-tau fibrils and 18S rRNA-tau fibrils were deposited in the Electron Microscopy Public Image Archive EMPIAR: EMPIAR-13150, EMPIAR-13153. Cryo-EM maps for AD-seeded ufRNA-tau fibrils and 18S rRNA-tau fibrils were deposited in the Electron Microscopy DataBank EMDB: EMD-70224, EMD-70227. Atomic models for AD-seeded ufRNA-tau fibrils and 18S rRNA-tau fibrils were deposited in the Protein DataBank PDB: 9o8e, 9o8h. RNA sequencing of RNA bound to AD-seeded ufRNA-tau fibrils was deposited in the Gene Expression Omnibus GEO: GSE319735.
• This paper does not report original code.
• Any additional information required to reanalyze the data reported in this paper is available from the lead contact upon request.

## Acknowledgments

We acknowledge support from the 10.13039/100000002National Institutes of Health grants R01AG070895 (D.S.E.), RF1AG065407 (D.S.E.), R01AI163216 (F.G.), and R21HD115071 (F.G.). We acknowledge support from the 10.13039/100000005Department of Defense grant HT94252410628. (F.G.). We thank Dr. Harry V. Vinters and Christopher K. Williams (UCLA Pathology) for providing postmortem AD brain tissue. We are grateful to Dr. Malcolm Roberts (Eisai) for sharing the 7G6 antibody and Dr. Virginia M.-Y. Lee (University of Pennsylvania) for sharing the GT-38 antibody. We are grateful to Dr. Marc I. Diamond (University of Texas Southwestern) for sharing tau biosensor cells. We thank Grant Shoffner for synthesizing RNAs. Cryo-EM data for AD-seeded ufRNA-tau fibrils were collected at the HHMI Janelia Research Campus CryoEM Facility. We thank Doreen Matthies, Momoko Shiozaki, and Zhiheng Yu for their help in the session. Cryo-EM data for unseeded 18S rRNA-tau fibrils were collected at the National Center for CryoEM Access and Training (NCCAT) and the Simons Electron Microscopy Center located at the New York Structural Biology Center, supported by 10.13039/100000002NIH (Common Fund U24GM129539, NIGMS R24GM154192), the 10.13039/100000893Simons Foundation (SF349247), and the NY State Assembly. We thank Jin Wang for his help in microscope operation and data collection. Figures created in BioRender (https://BioRender.com).

## Author contributions

R.A., J.Z., and J.L.D. produced tau proteins, extracted mouse liver RNA, formed fibrils, and performed biochemical characterization of fibrils. J.P. performed gel shift assays. R.A. performed RNA sequencing. R.A., Y.X.J., P.G., D.R.B., and D.C. collected and processed cryo-EM data. R.A., Y.X.J., and M.R.S. analyzed and illustrated fibril structures. R.A., Y.X.J., M.R.S., and D.S.E. wrote the manuscript with contributions from all authors. D.S.E. and F.G. supervised the project.

## Declaration of interests

D.S.E. is an advisor and equity shareholder in ADRx, Inc.

## STAR★Methods

### Key resources table

**Table:** 

REAGENT or RESOURCE	SOURCE	IDENTIFIER
**Antibodies**		

Mouse anti-tau (clone 43D)	BioLegend	Cat#816601
Mouse anti-tau (clone 7G6)	Malcolm Roberts, Eisai (Roberts et al.^21^)	N/A
Mouse anti-tau (clone Tau46)	BioLegend	Cat#806604
Mouse anti-tau (clone GT-38)	Virginia M.-Y. Lee, University of Pennsylvania (Gibbons et al.^23^)	N/A
Rabbit anti-tau	Dako	Cat#A0024
6 nm Colloidal Gold AffiniPure Goat Anti-Mouse IgG	Jackson ImmunoResearch	Cat#115-195-166
Goat Anti-Mouse HRP	Abcam	Cat#ab205719
Goat Anti-Rabbit HRP	Thermo Fisher Scientific	Cat#31460

**Bacterial and virus strains**		

*Escherichia coli* BL21(de3) Gold Competent Cells	Agilent	Cat#230132

**Biological samples**		

Alzheimer’s disease patient (86-year-old female) left temporal lobe brain tissue	UCLA Pathology	Case#3-164

**Chemicals, peptides, and recombinant proteins**		

Dulbecco’s Modified Eagle Medium	Thermo Fisher Scientific	Cat#11965092
Fetal Bovine Serum	Thermo Fisher Scientific	Cat#A3160401
Antibiotic-Antimycotic	Thermo Fisher Scientific	Cat#15240062
GlutaMAX Supplement	Thermo Fisher Scientific	Cat#35050061
Halt Protease Inhibitor Cocktail	Thermo Fisher Scientific	Cat#87786
TRIzol Reagent	Thermo Fisher Scientific	Cat#15596018
N-lauroylsarcosine sodium salt	Millipore Sigma	Cat#L5125
Lipofectamine 3000	Thermo Fisher Scientific	Cat#11668027
Opti-MEM	Thermo Fisher Scientific	Cat#31985062
2% Uranyl Acetate Solution	Electron Microscopy Sciences	Cat#22400-2
Blocking-Grade Blocker	Bio-Rad	Cat#1706404
Pierce ECL Plus Western Blotting Substrate	Thermo Fisher Scientific	Cat#32132
RNase A	Thermo Fisher Scientific	Cat#EN0531
Heparin sodium salt	Sigma	Cat#H3393
SYBR Green II RNA Gel Stain	Thermo Fisher Scientific	Cat#S7564
Orange SYPRO Protein Gel Stain	Thermo Fisher Scientific	Cat#S6650

**Critical commercial assays**		

QIAquick Gel Extraction Kit	Qiagen	Cat#28704

**Deposited data**		

Cryo-EM micrographs of AD-seeded ufRNA-tau fibrils	This paper	EMPIAR-13150
Cryo-EM micrographs of unseeded 18S rRNA-tau fibrils	This paper	EMPIAR-13153
Cryo-EM map of AD-seeded ufRNA-tau fibrils	This paper	EMD-70224
Cryo-EM map of unseeded 18S rRNA-tau fibrils	This paper	EMD-70227
Atomic model of AD-seeded ufRNA-tau fibrils	This paper	PDB 9o8e
Atomic model of unseeded 18S rRNA-tau fibrils	This paper	PDB 9o8h
RNA sequencing of RNA bound to AD-seeded ufRNA-tau fibrils	This paper	GSE319735

**Experimental models: Cell lines**		

HEK296T tau biosensor cells	Marc I. Diamond, University of Texas Southwestern (Holmes et al.^20^)	N/A

**Experimental models: Organisms/strains**		

C57BL/6J mice liver tissue	The Jackson Laboratory	N/A

**Recombinant DNA**		

pET28b-Tau40-6xHis	This paper	N/A

**Software and algorithms**		

SerialEM	Mastronarde^39^	https://bio3d.colorado.edu/SerialEM/
Leginon	Suloway et al.^40^	https://leginon-org.github.io/leginon/leginon/
CTFFIND4	Rohou and Grigorieff^41^	https://grigoriefflab.umassmed.edu/ctffind4
Unblur	Grant and Grigorieff^42^	https://grigoriefflab.umassmed.edu/unblur_summovie
MotionCorr	Li et al.^43^	https://emcore.ucsf.edu/ucsf-software
EMAN2	Tang et al.^44^	https://blake.bcm.edu/emanwiki/doku.php?id=eman2
crYOLO	Wagner et al.^45^	https://cryolo.readthedocs.io/en/stable/
RELION3	He and Scheres^46^	https://www2.mrc-lmb.cam.ac.uk/relion
phenix.auto_sharpen	Terwilliger et al.^47^	https://phenix-online.org/
Coot	Emsley et al.^48^	https://www2.mrc-lmb.cam.ac.uk/personal/pemsley/coot/
phenix.real_space_refine	Afonine et al.^49^	https://phenix-online.org/
phenix.comprehensive_validation	Afonine et al.^50^	https://phenix-online.org/
MolProbity	Williams et al.^51^	http://molprobity.biochem.duke.edu/
PyMOL	Schrödinger LLC^52^	https://www.pymol.org/
bcl2fastq v2.19.1.403	Illumina	https://support.illumina.com/sequencing/sequencing_software/bcl2fastq-conversion-software.html
Partek Flow	Illumina	https://www.illumina.com/products/by-type/informatics-products/partek-flow.html
STAR v2.7.9a	Dobin et al.^53^	https://software.cqls.oregonstate.edu/updates/star-2.7.9a/
ImageJ	Schneider et al.^54^	https://imagej.net/ij/
GraphPad Prism	Prism v10.0.0	https://www.graphpad.com
Adobe Photoshop	Photoshop 26.6.0	https://www.adobe.com/products/photoshop.html

**Other**		

5 mL HisTrap HP His tag protein purification column	Cytiva	Cat#17524801
5 mL HiTrap SP HP cation exchange chromatography column	Cytiva	Cat#17115101
120 mL Superdex 200 prep grade SEC resin in HiLoad column	Cytiva	Cat#28989335
Amicon Ultra Centrifugal Filter 10 kDa MWCO	MilliporeSigma	Cat#UFC901024
Polytron PT 10-35	Kiematica	N/A
SC20 Digital Orbital Mixing/Heating Dry Bath	Torrey Pines Scientific	N/A
Celigo Image Cytometer	Nexcelom	Cat#200-BFFL-5C
Axio Observer D1 Inverted Fluorescence Microscope	Zeiss	Cat#431006
400 mesh carbon-coated formvar support films mounted on copper grids	Ted Pella, Inc.	Cat#01754-F
Quantifoil R 1.2/1.3 Holey Carbon Films on Grids 200 Mesh, Cu	Electron Microscopy Sciences	Cat#Q2100CR1.3
NuPAGE Bis-Tris Mini Protein Gels 4–12% 1.5 mm	Thermo Fisher Scientific	Cat#NP0336BOX
Nitrocellulose Membrane 0.2 μm	Bio-Rad	Cat#1620146
Azure Imaging Systems 600	Azure Biosystems	Cat#AZI600-01
NanoDrop Spectrophotometer	Thermo Fisher Scientific	Cat#ND2000
Typhoon FLA 9500	GE	Cat#29-0044-13
DNA/RNA Synthesizer	K&A Labs GmbH	N/A

### Experimental model and study participant details

#### Postmortem Alzheimer’s disease brain tissue

Frozen autopsied human brain tissue was obtained from UCLA Pathology operating under protocols approved by the UCLA Institutional Review Board and according to US Department of Health and Human Services regulations. The left temporal lobe of an 86-year-old female neuropathologically diagnosed with Braak stage IV Alzheimer’s disease (case ID 3–164) was used in this study.

#### HE293T tau biosensor cells

A HEK293T cell line stably expressing tau K18 (residues 244–372) with P301S mutation fused to YFP or CFP were generated and kindly shared by Dr. Marc I. Diamond (University of Texas Southwestern). The parental cell line was authenticated by ATCC and tested negative for mycoplasma. Cells were maintained in Dulbecco’s Modified Eagle Medium (Thermo Fisher Scientific, Cat. No. 11965092) supplemented with 10% (v/v) Fetal Bovine Serum (Thermo Fisher Scientific, Cat. No. A3160401), 1% Antibiotic-Antimycotic (Thermo Fisher Scientific, Cat. No. 15240062) and 1% GlutaMAX Supplement (Thermo Fisher Scientific, Cat. No. 35050061) at 37 °C, 5% CO_2_ in a humidified incubator.

### Method details

#### Expression and purification of tau proteins

Full-length human 2N4R tau residues 1–441 (tau40) was expressed and purified as previously described.^55^^,^^56^^,^^57^ Tau40 with a C-terminal his-tag (MAEPRQEFEVMEDHAGTYGLGDRKDQGGYTMHQDQEGDTDAGLKESPLQTPTEDGSEEPGSETSDAKSTPTAEDVTAPLVDEGAPGKQAAAQPHTEIPEGTTAEEAGIGDTPSLEDEAAGHVTQARMVSKSKDGTGSDDKKAKGADGKTKIATPRGAAPPGQKGQANATRIPAKTPPAPKTPPSSGEPPKSGDRSGYSSPGSPGTPGSRSRTPSLPTPPTREPKKVAVVRTPPKSPSSAKSRLQTAPVPMPDLKNVKSKIGSTENLKHQPGGGKVQIINKKLDLSNVQSKCGSKDNIKHVPGGGSVQIVYKPVDLSKVTSKCGSLGNIHHKPGGGQVEVKSEKLDFKDRVQSKIGSLDNITHVPGGGNKKIETHKLTFRENAKAKTDHGAEIVYKSPVVSGDTSPRHLSNVSSTGSIDMVDSPQLATLADEVSASLAKQGLLEHHHHHH) was expressed in pET28b using BL21-Gold Competent *Escherichia coli* cells (Agilent, Cat. No. 230132) grown in TB medium to OD600–0.8. Protein expression was induced with 1 mM IPTG at 37 °C for 3 h, then cells were lysed by sonication in lysis buffer: 50 mM Tris pH 8.0, 500 mM NaCl, 20 mM imidazole, 1 mM beta-mercaptoethanol and Halt Protease Inhibitor Cocktail (Thermo Fisher Scientific, Cat. No. 87786). Cell lysate was centrifuged at 15,000 rpm for 15 min, then the supernatant loaded onto a 5 mL HisTrap HP His tag protein purification column (Cytiva, Cat. No. 17524801). The column was washed with lysis buffer and eluted over an imidazole gradient from 20 mM to 300 mM. Fractions containing tau40 were dialyzed into 50 mM MES pH 6.0, 50 mM NaCl and 1 mM beta-mercaptoethanol, then purified on a 5 mL HiTrap SP HP cation exchange chromatography column (Cytiva, Cat. No. 17115101). Tau40 fractions were purified by size exclusion chromatography using a 120 mL Superdex 200 prep grade SEC resin in HiLoad column (Cytiva, Cat. No. 28989335) in PBS pH 7.4, then concentrated to 20–60 mg/mL using Amicon Ultra Centrifugal Filter 10 kDa MWCO (MilliporeSigma, Cat. No. UFC901024).

#### Isolation of mouse liver RNA

Liver tissue harvested from 10 to 12 week old male C57BL/6J mice was purchased from The Jackson Laboratory. 100 mg of tissue was homogenized in 1 mL TRIzol Reagent (Thermo Fisher Scientific, Cat. No. 15596018), followed by RNA extraction with 0.2 mL chloroform. Unfractionated RNA was precipitated using 0.5 mL isopropanol, then resuspended in 100 μL RNase-free water. Unfractionated RNA was subjected to electrophoresis on 1% agarose gel at 110 V for 40 min, which yielded two major bands corresponding to 28S and 18S ribosomal RNA. 18S ribosomal RNA bands were excised and extracted using a QIAquick Gel Extraction Kit (Qiagen, Cat. No. 28704).

#### Extraction of tau fibrils from AD brain

*Ex vivo* tau fibrils from postmortem brain of an AD patient was extracted as previously described.^58^ Briefly, frozen tissue was homogenized using a Polytron PT 10–35 (Kiematica) in 10 mM Tris-HCl pH 7.4, 0.8 M NaCl, 10% (w/v) sucrose supplemented with 1 mM EGTA, then centrifuged at 20,000 *g* at 4 °C for 20 min. The supernatant incubated with 1% (w/v) N-lauroylsarcosine sodium salt (sarkosyl, Millipore Sigma, Cat. No. L5125) at 22 °C with 190 rpm shaking for 1 h, then centrifuged at 100,000 *g* at 4 °C for 1 h. The pellet was resuspended in 10 mM Tris-HCl pH 7.4, 0.8 M NaCl, 5 mM EDTA, 1 mM EGTA, 10% (w/v) sucrose, then centrifuged at 20,100 *g* at 4 °C for 30 min. The supernatant was centrifuged at 65,000 *g* at 22 °C for 1 h. The sarkosyl-insoluble pellet, enriched with tau fibrils, was resuspended in 20 mM Tris-HCl pH 7.4, 100 mM NaCl.

#### Formation of RNA-tau fibrils

For AD-seeded ufRNA-tau fibrils, purified recombinant tau40 was diluted to 50 μM in 20 mM ammonium acetate pH 7.0 and incubated with 400 μg/mL of unfractionated mouse liver RNA and 5% (v/v) AD tau fibrils with ∼1000 rpm shaking in an SC20 Digital Orbital Mixing/Heating Dry Bath (Torrey Pines Scientific) at 37 °C for 2 days. First generation fibrils were clumped and were used to seed tau40 with unfractionated mouse liver RNA and AD tau fibrils, as described before. Second generation fibrils were used for biochemical and cryo-EM study. For unseeded 18S rRNA-tau fibrils, purified recombinant tau40 was diluted to 50 μM in 20 mM ammonium acetate pH 7.0 and incubated with 50 μg/mL of mouse liver 18S ribosomal RNA with ∼1000 rpm shaking in an SC20 Digital Orbital Mixing/Heating Dry Bath at 37 °C for 3 days. Fibrils were centrifuged at 120,000 *g* for 1 h, resuspended in 10X concentrated volume of 20 mM Tris-HCl pH 7.5 and 150 mM NaCl for cryo-EM.

#### Tau biosensor cell seeding

RNA-tau fibrils or AD tau fibrils were sonicated in a cuphorn ice water bath for 5 min, then mixed with equivolume of Lipofectamine 3000 (Thermo Fisher Scientific, Cat. No. 11668027) prepared by diluting 1 μL in 19 μL of Opti-MEM (Thermo Fisher Scientific, Cat. No. 31985062). After 20 min, 10 μL of fibril-lipofectamine mixture was added to 90 μL of tau biosensor cells. After 3 days, triplicates of 96-well plate wells were imaged using a Celigo Image Cytometer (Nexcelom, Cat. No. 200-BFFL-5C) in the YFP channel for quantification. For figures, cells were imaged using an Axio Observer D1 Inverted Fluorescence Microscope (Zeiss, Cat. No. 431006) in the YFP channel.

#### Negative stain TEM

400 mesh carbon-coated formvar support films mounted on copper grids (Ted Pella, Inc., Cat. No. 01754-F) were glow discharged for 30 s 4 μL of sample was applied onto grids, incubated for 1 min and blotted off with filter paper. 4 μL of 2% Uranyl Acetate Solution (Electron Microscopy Sciences, Cat. No. 22400-2) was applied onto grids, incubated for 2 min and blotted off with filter paper. Grids were washed with another 4 μL of 2% uranyl acetate and air-dried. Grids were imaged using a Tecnai T12 Transmission Election Microscope (FEI).

#### Immunogold labeling

400 mesh carbon-coated formvar support films mounted on cooper grids were glow discharged for 30 s 5 μL of fibril sample was applied onto grids, incubated for 3 min and blotted off with filter paper. Grids were blocked with 0.1% gelatin in PBS for 10 min, then incubated with 43D (BioLegend, Cat. No. 816601), 7G6 (kindly shared by Dr. Malcolm Roberts, Eisai), Tau46 (BioLegend, Cat No: 806604) primary antibodies at a dilution of 1:1000 in 0.1% gelatin in PBS for 1 h, except Tau46 at a dilution of 1:50. Grids were incubated with 6 nm Colloidal Gold AffiniPure Goat Anti-Mouse IgG (Jackson ImmunoResearch, Cat. No. 115-195-166) at a dilution of 1:8 in 0.1% gelatin in PBS for 30 min. Grids were washed five times with water, stained with 4 μL of 2% Uranyl Acetate Solution (Electron Microscopy Sciences, Cat. No. 22400-2) and air-dried. Grids were imaged using a Tecnai T12 Transmission Election Microscope (FEI).

#### Western blot

RNA-tau fibrils were mixed with SDS-PAGE loading dye containing 2 M urea and subjected to electrophoresis on NuPAGE Bis-Tris Mini Protein Gels 4–12% 1.5 mm (Thermo Fisher Scientific, Cat. No. NP0336BOX) at 200 V for 30 min, then transferred onto Nitrocellulose Membrane 0.2 μm (Bio-Rad, Cat. No. 1620146). Membranes were blocked in 5% (w/v) Blocking-Grade Blocker (Bio-Rad, Cat. No. 1706404) in Tris-buffered saline with 0.1% (v/v) Tween 20 (TBST) at room temperature for 1 h. Membranes were immunoblotted using primary antibodies 43D (BioLegend, Cat. No. 816601) at a 1:5000 dilution, 7G6 (kindly shared by Dr. Malcolm Roberts, Eisai) at a 1:5000 dilution, and Tau46 (BioLegend, Cat No: 806604) at a 1:1000 dilution in TBST with 2% (w/v) Blocker at room temperature for 1 h. After three TBST washes, membranes were incubated with Goat Anti-Mouse HRP secondary antibody (Abcam, Cat. No. ab205719) at a dilution of 1:4000 in TBST with 2% (w/v) Blocker at room temperature for 1 h. After three TBST washes, membranes were developed using Pierce ECL Plus Western Blotting Substrate (Thermo Fisher Scientific, Cat. No. 32132) and imaged using an Azure Imaging Systems 600 (Azure Biosystems, Cat. No. AZI600-01).

#### Dot blot

Unseeded 18S rRNA-tau fibrils and other recombinant tau samples were applied onto Nitrocellulose Membrane 0.2 μm (Bio-Rad, Cat. No. 1620146). Membranes were blocked in 5% (w/v) Blocking-Grade Blocker (Bio-Rad) in Tris-buffered saline with 0.1% (v/v) Tween 20 (TBST) at room temperature for 1 h. Membranes was immunoblotted using primary antibodies GT-38 (kindly provided by Dr. Virginia M.-Y. Lee, University of Pennsylvania) at a 1:1000 dilution, and Dako (Dako, Cat. No. A0024) at a 1:4000 dilution in TBST with 2% (w/v) Blocker at room temperature for 2 h. After three TBST washes, membranes were incubated with Goat Anti-Mouse HRP-conjugated secondary antibody (Abcam, Cat. No. ab205719) or Goat Anti-Rabbit HRP-conjugated secondary antibody (Thermo Fisher Scientific, Cat. No. 31460) at a 1:4000 dilution in TBST with 2% (w/v) Blocker at room temperature for 1 h. After three TBST washes, membranes were developed using Pierce ECL Plus Western Blotting Substrate (Thermo Fisher Scientific, Cat. No. 32132) and imaged using an Azure Imaging Systems 600 (Azure Biosystems, Cat. No. AZI600-01).

#### RNase treatment of tau fibrils

RNA-tau fibrils were centrifuged at 90,000 rpm for 1 h and washed twice with RNase-free water. Fibrils were treated with 1:0, 1:0.1, 1:1 and 1:10 M ratios of fibril:RNase A (Thermo Fisher Scientific, Cat. No. EN0531) in 20 mM ammonium acetate pH 7.0 at 37 °C for 2 h. AD tau fibrils were diluted 5x from their frozen concentration and treated with 0, 0.1, 1, and 10 μg/μL RNase A at 37°C overnight.

#### Spectroscopic detection of fibril-bound RNA

Heparin-tau fibrils were prepared by incubating 25 μM tau40, 0.225 mg/mL Heparin sodium salt (Sigma, Cat. No. H3393) and 1 mM DTT in PBS buffer pH 6.8 with ∼1000 rpm shaking in an SC20 Digital Orbital Mixing/Heating Dry Bath (Torrey Pines Scientific) at 37 °C for 6 days. RNA-tau fibrils and heparin-tau fibrils were centrifuged at 90,000 rpm for 1 h and washed twice with RNase-free water. Samples were diluted in RNase-free water to equimolar concentrations of tau and RNA. Absorption spectra from 220 to 350 nm were measured using a NanoDrop Spectrophotometer (Thermo Fisher Scientific, Cat. No. ND2000).

#### Electrophoretic mobility shift assay

100 nM RNA and 1:1.25, 1:2.5, 1:5 M ratios of RNA:tau40 were mixed in 50 mM Tris pH 8.0, 150 mM KCl, 0.01% Tween 20 and incubated on ice for 1 h 1 μL of 50% glycerol was added to 10 μL samples, which were subjected to electrophoresis on 0.8% agarose gels at 65 V for 75 min, in chilled 1X TBE buffer in a gel apparatus surrounded with ice. The gel was incubated with 1X SYBR Green II RNA Gel Stain (Thermo Fisher Scientific, Cat. No. S7564) for 30 min and visualized using a Typhoon FLA 9500 (GE, Cat. No. 29-0044-13). Subsequently, the gel was incubated with 0.05% SDS, 7.5% acetic acid and 5X Orange SYPRO Protein Gel Stain (Thermo Fisher Scientific, Cat. No. S6650) overnight and visualized for protein. The 20mer hairpin (18S rRNA 1840–1861) and poly A oligos were synthesized using a DNA/RNA Synthesizer (K&A Labs GmbH) and purified via high-performance liquid chromatography. 20mer hairpin and poly A with tau40 samples were subjected to electrophoresis on a 15% polyacrylamide native gel at 100 V for 60 min, then visualized the same way.

#### Cryo-EM

2.5 μL of RNA-tau fibril sample were applied to Quantifoil R 1.2/1.3 Holey Carbon Films on Grids 200 Mesh, Cu (Electron Microscopy Sciences, Cat. No. Q2100CR1.3) glow discharged for 4 min. Grids were blotted with filter paper and plunge frozen in liquid ethane using a Vitrobot Mark IV (FEI). Cryo-EM data was collected on a Titan Krios (FEI) microscope with a K3 Camera (Gatan), operated at 300 kV with a 20 eV energy filter. For AD-seeded ufRNA-tau fibrils, automated data collection was driven by SerialEM.^39^ Anisotropic magnification distortion estimation, CTF estimation and beam-induced motion correction were performed using mag-distortion-estimate,^59^ CTFFIND4^41^ and Unblur,^42^ respectively. The physical pixel size was corrected to 1.077 Å/pixel after anisotropic magnification correction with Unblur. For unseeded 18S rRNA-tau fibrils, automated data collection was driven by Leginon.^40^ Motion correction and CTF estimation were performed using MotionCorr^43^ and CTFFIND4,^41^ respectively.

#### Helical reconstruction

Particles were picked manually using EMAN2 e2helixboxer.py.^44^ For unseeded 18S rRNA-tau fibrils, manually-picked particles from 200 micrographs were used to train automated particle picking using crYOLO.^45^^,^^60^ Particle extraction, 2D classification, helical reconstruction and 3D refinement were performed using RELION3.^46^^,^^61^^,^^62^ For AD-seeded ufRNA-tau fibrils, particles were extracted using 686-pixel box size binned by 2 with 68.6-pixel inter-box or 320-pixel box size with 32-pixel inter-box. For unseeded 18S rRNA-tau fibrils, particles were extracted using 640-pixel box size binned by 2 or 320-pixel box size with 32-pixel inter-box. 2D classification was performed on 686-pixel box or 640-pixel box particles to estimate the fibril pitch and helical parameter, as well as generate initial models. Helical reconstruction was performed on 320-box particles with a 220 Å outer helical mask, manually controlling the tau2_fudge and healpix_order parameters. 3D classification was performed to discard poor particles, then helical refinement was performed on the optimized subset of particles. Contrast transfer function refinement, Bayesian polishing and golden-standard 3D auto-refinement were performed. Refined maps were sharpened using phenix.auto_sharpen^47^ at the 0.143 resolution cutoff of half map Fourier shell correlations. Cryo-EM data collection and processing statistics are summarized in Table 1. RNA-tau fibrils are presumed to be left-handed, as are the majority of amyloid fibrils.

#### Atomic model building

Atomic model building was performed using Coot.^48^ Five-layer models were generated via the helical parameters and iteratively refined using phenix.real_space_refine.^49^ Final models were validated using phenix.comprehensive_validation^50^ and MolProbity.^51^ Model composition and validation statistics are summarized in Table 1. Structures illustrated using PyMOL.^52^

#### Solvation energy calculation

Solvation energy was calculated for each residue by the sum of the products of the area buried for each atom and the corresponding atomic solvation parameter, as described previously.^63^ Residues were colored by their calculated energy. The overall energies per chain are the sums of energies of all residues. To account for charge compensation by RNA, energetic penalties were removed for lysine primary amine nitrogens (1 per residue), arginine guanidino nitrogens (3 per residue) and histidine imidazole nitrogens (2 per residue) for residues in contact with residual density in the cryo-EM map.

#### RNA sequencing

AD-seeded ufRNA-tau fibrils were centrifuged at 100,000 rpm for 1 h and washed twice with RNase-free water. RNA sequencing was performed at the UCLA Technology Center for Genomics & Bioinformatics on a NovaSeq 6000 (Illumina) using a 2 × 150 bp paired-end run and assessed with Sequencing Analysis Viewer (Illumina). Demultiplexing was performed using bcl2fastq v2.19.1.403 (Illumina). Reads were aligned using STAR v2.7.9a^53^ with default parameters and read counts per gene were quantified using mouse genome mm39 in Partek Flow (Illumina).

### Quantification and statistical analysis

#### Tau biosensor cell seeding

Seeded aggregates were quantified by counting fluorescence peaks above background with the Particle Analyzer tool in ImageJ.^54^ Number of aggregates in was normalized to confluence of each well. Average and standard deviation values were calculated from triplicate well measurements and plotted using GraphPad Prism 10.0.0.

## Supplemental information

Document S1. Figures S1–S10

Table S1. Tau fibril structure solvation energies tabulated from Amyloid Atlas (https://people.mbi.ucla.edu/sawaya/amyloidatlas/) on March 9, 2025
