# Supplementary material for: Structural evidence that RNA contributes to polymorphism of tau amyloid fibrils
Source: iScience. 2026 Mar 26;29(4):115501. doi: 10.1016/j.isci.2026.115501 (PMC13091454; doi:10.1016/j.isci.2026.115501)
Supplement: Document S1. Figures S1–S10 [file mmc1.pdf]

iScience, Volume *29*

Supplemental information

Structural evidence that RNA contributes to polymorphism of tau amyloid fibrils

Romany Abskharon, Yi Xiao Jiang (江逸潇), Michael R. Sawaya, Peng Ge, Jeffrey Zhang, David R. Boyer, Joshua L. Dolinsky, Justin Pi, Duilio Cascio, Feng Guo (郭峰), and David S. Eisenberg

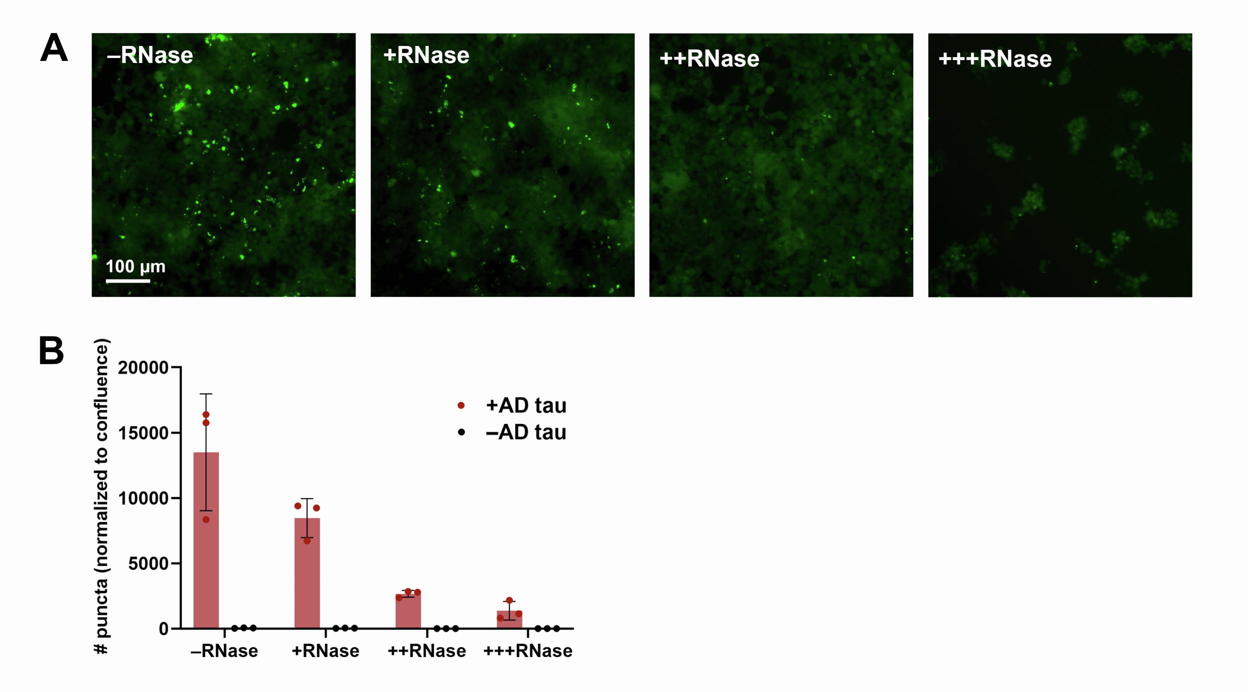

Figure S1. RNase A treatment abolishes biosensor cell seeding of AD tau fibrils. A) Confocal images and **B)** quantification of tau biosensor cell seeding by tau fibrils extracted from postmortem brain of an AD patient (AD tau) treated with 0 µg/µL (–RNase), 0.1 µg/µL (+RNase), 1 µg/µL (++RNase), and 10 µg/µL RNase A (+++RNase). Scale bar 100 µm. Data are represented as mean +/- SD.

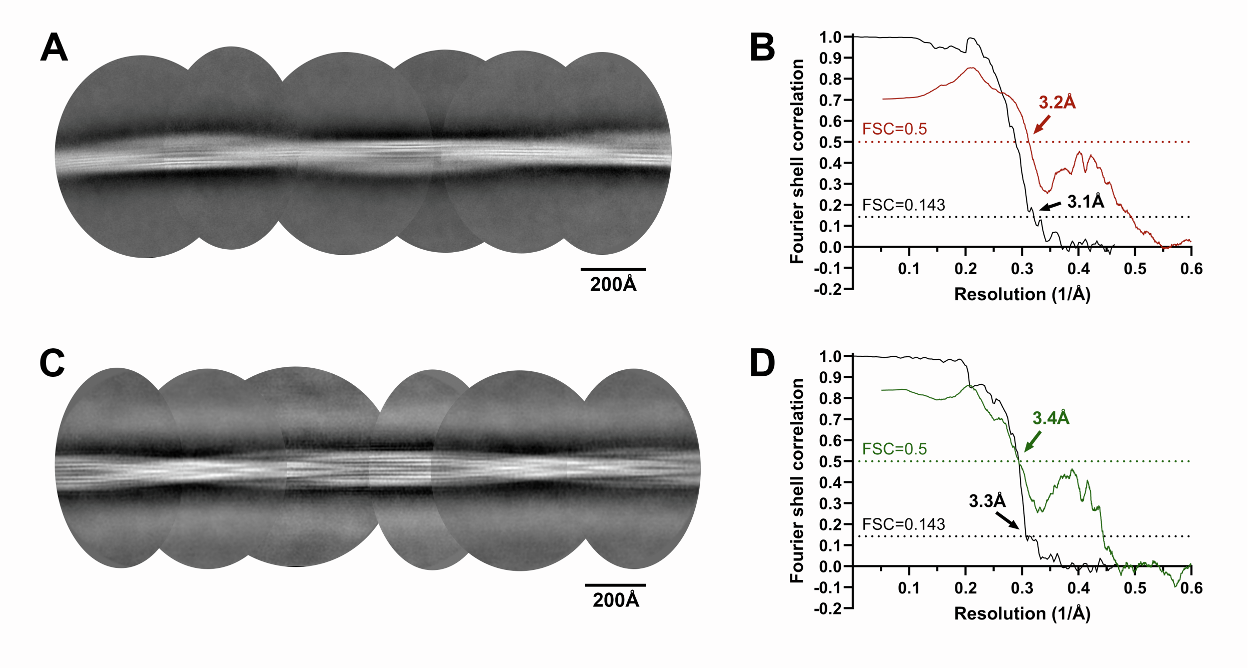

Figure S2. Cryo-EM 2D classes and FSC curves of RNA-tau fibrils. A) Stitched cryo-EM 2D classes of 686-pixel box particles and **B)** Fourier shell correlation curves for two independent half-maps (black) and the refined atomic model against the cryo-EM map (red) of AD-seeded ufRNA-tau fibril. **C)** Stitched cryo-EM 2D classes of 640-pixel box particles and **D)** Fourier shell correlation curves for two independent half-maps (black) and the refined atomic model against the cryo-EM map (green) of the unseeded 18S rRNA-tau fibril. FSC thresholds of 0.143 (black dashed line) and 0.5 (red and green dashed lines) are shown. Scale bar 200 Å.

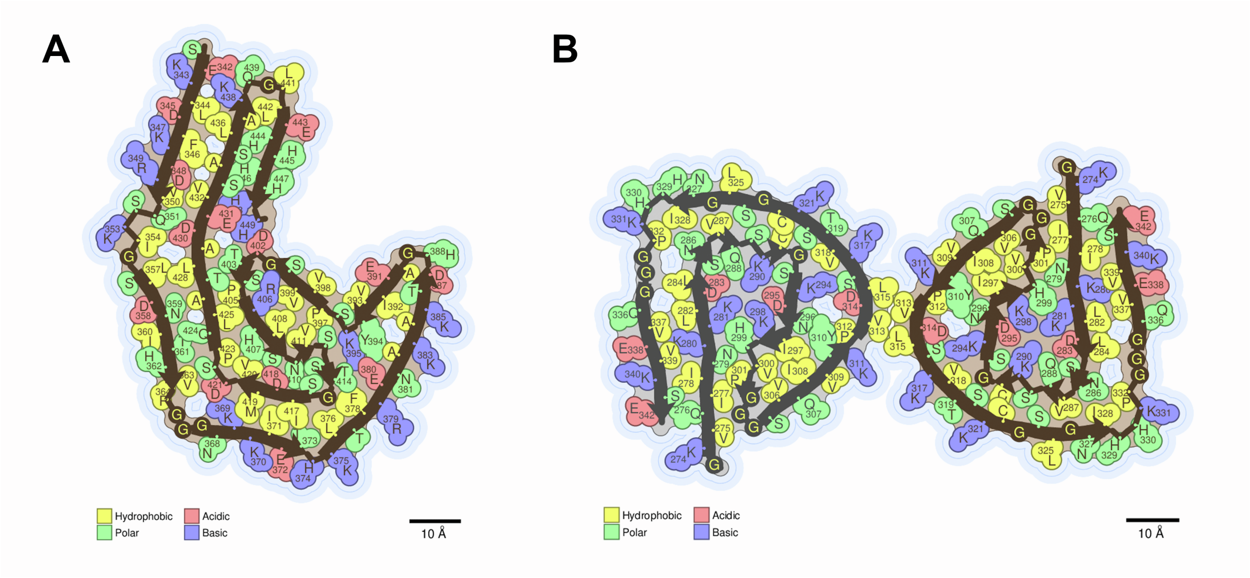

Figure S3. Polarity maps of RNA-tau fibrils. Polarity maps of **A)** AD-seeded ufRNA-tau fibril and **B)** unseeded 18S rRNA-tau fibril. Scale bar 10 Å.

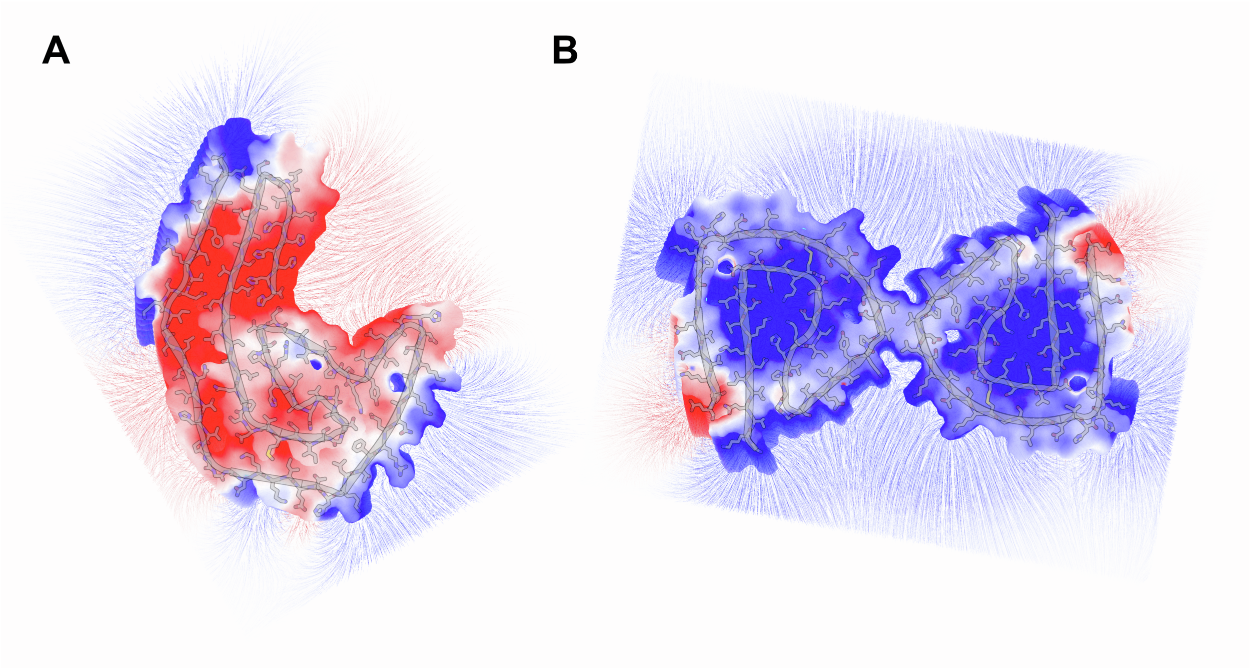

Figure S4. Surface electrostatic potential of RNA-tau fibrils. The surface of **A)** AD-seeded ufRNA-tau fibril and **B)** unseeded 18S rRNA-tau fibril colored by positive (blue) and negative (red) electrostatic potential.

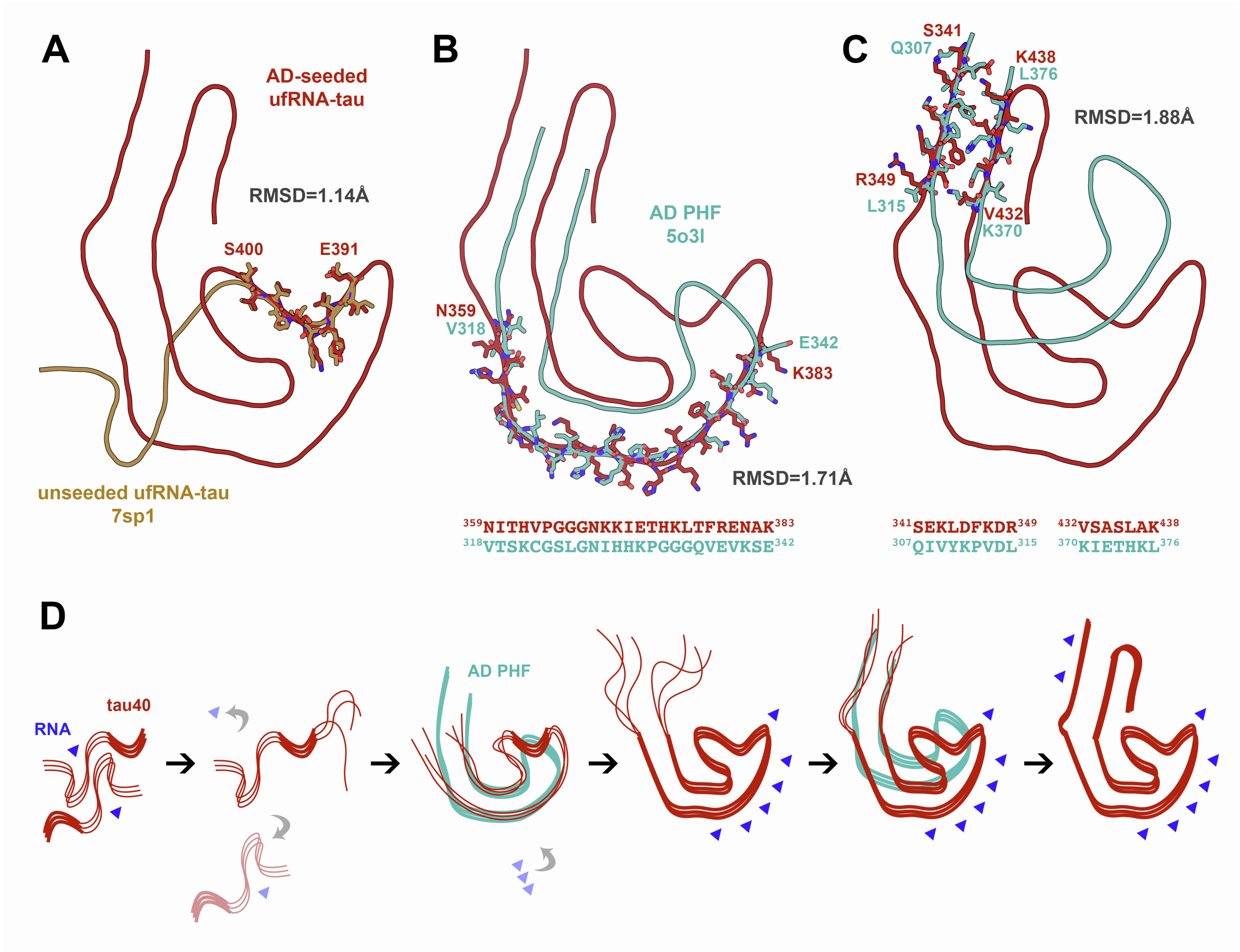

Figure S5. AD tau templates segments of its C-shaped fold onto ufRNA-tau fibrils. AD-seeded ufRNA-tau fibril (red) superimposed with **A)** unseeded ufRNA-tau fibril (yellow, PDB 7sp1) for E391 to S400, **B)** AD PHF fold (teal, PDB 5o3l) for AD-seeded ufRNA-tau fibril N359 to K383 with AD PHF V318 to E342, and **C)** AD-seeded ufRNA-tau fibril S341 to R349 and V432 to K438 with AD PHF Q307 to L315 and K370 to L376. **D)** Schematic of tau40 (red) fibril formation in the presence of RNA (blue triangles) seeded by AD PHF (teal).

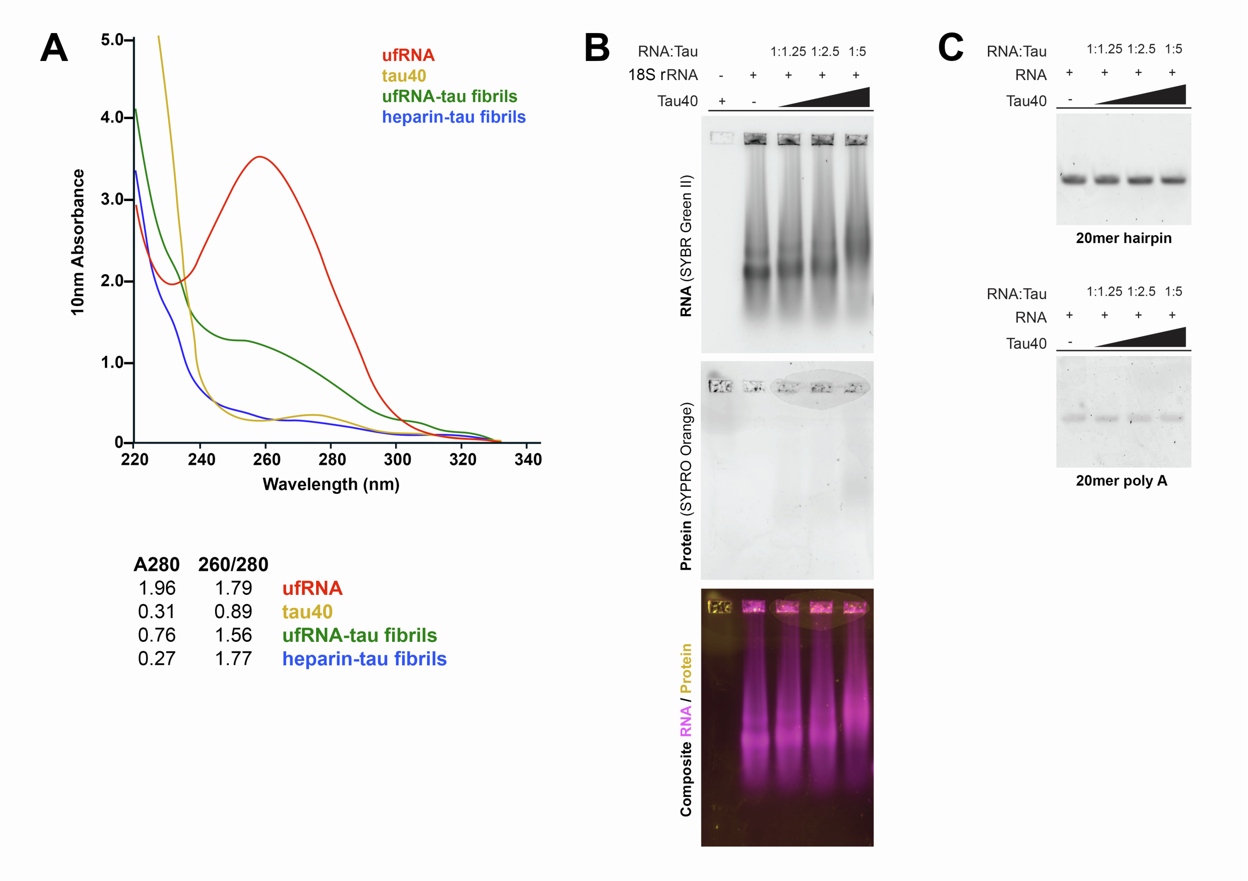

Figure S6. Spectrometric and gel shift evidence of RNA in complex with tau fibrils. A) Absorption spectra for fibril-bound RNA of unseeded ufRNA-tau fibrils, with ufRNA, tau40 and heparin-tau fibrils as controls. A280 and A260/A280 values are shown. **B)** Electrophoretic mobility shift assay of tau40 and mouse liver 18S rRNA with 1:1.25, 1:2.5 and 1:5 molar ratios of RNA:tau40. **C)** Electrophoretic mobility shift assay of a 20mer hairpin corresponding to 18S rRNA 1840-1861 (top) and polyA (bottom) with tau40 at 1:1.25, 1:2.5 and 1:5 molar ratios of RNA:tau40.

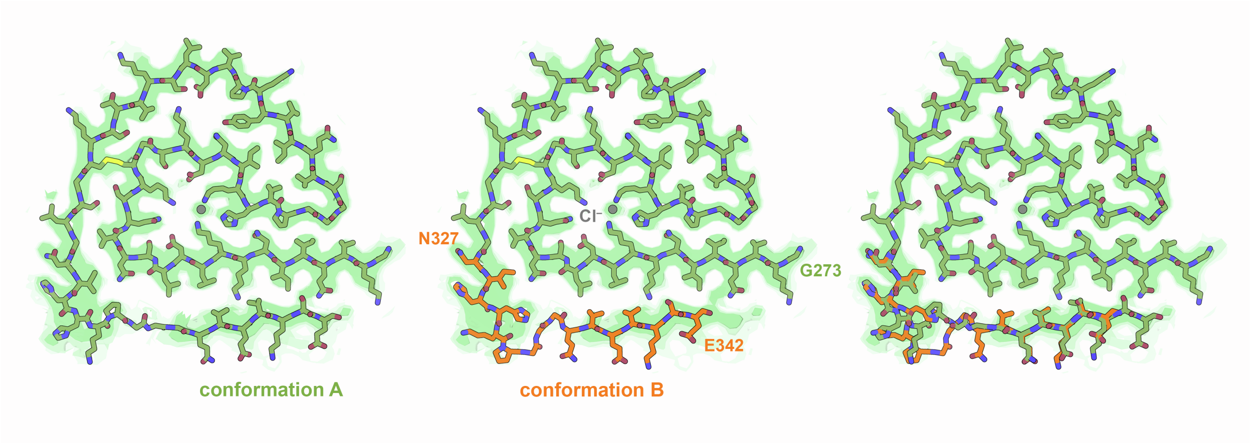

Figure S7. Alternative conformations of unseeded 18S rRNA-tau fibril for residues N327 to E342.

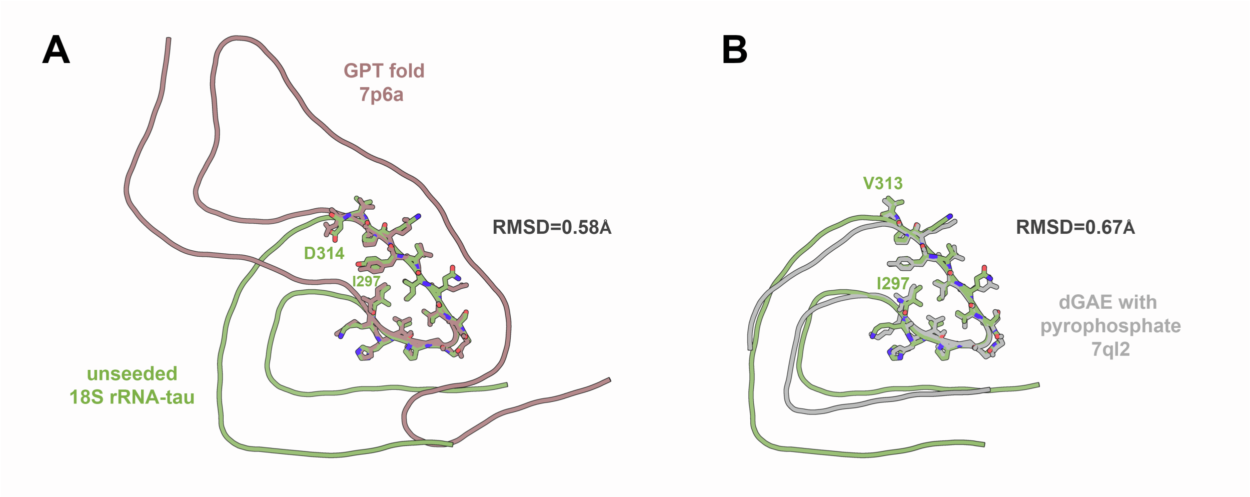

Figure S8. Similarity of unseeded 18S rRNA-tau fibril to GPT fold and recombinant dGAE fibril formed with pyrophosphate. Unseeded 18S rRNA-tau fibril protofilament (green) superimposed with **A)** GGT-PSP-Tau (GPT) fold (brown, PDB 7p6a) for residues I297 to D314, and with **B)** recombinant dGAE fibril formed with pyrophosphate (gray, PDB 7ql2) for residues I297 to V313.

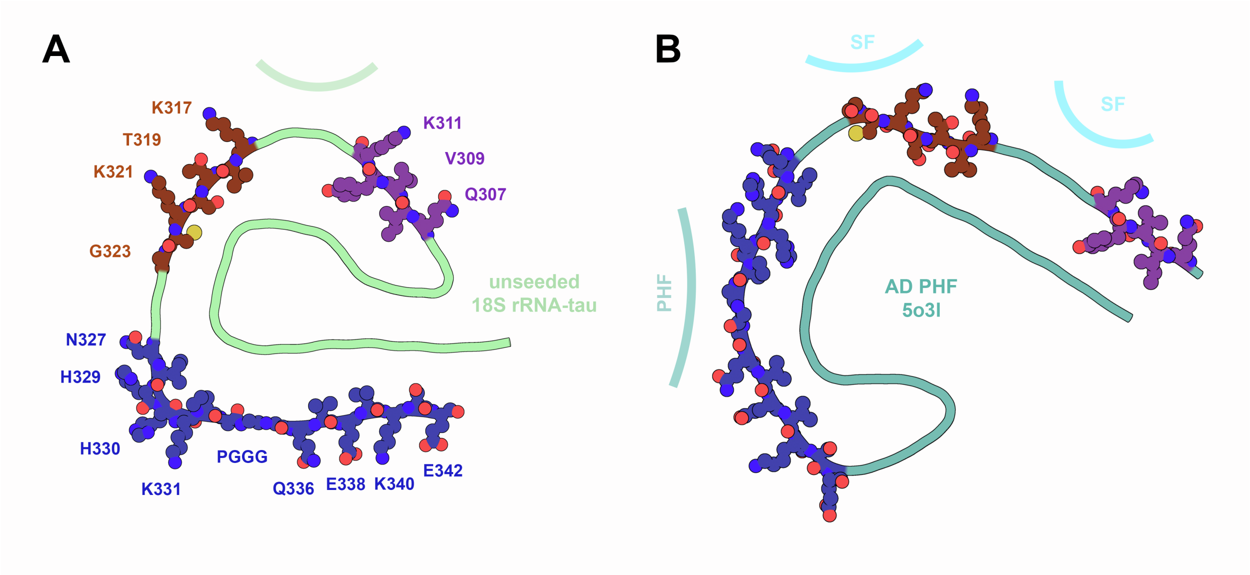

Figure S9. Shared structural motifs in unseeded 18S rRNA-tau fibril and AD PHF cores suggest potential amyloid epitopes of GT38 antibody. Solvent accessible segments Q307 to K311 (purple), K317 to G323 (brown), N327 to E342 (K329 (blue) are highlighted on **A)** unseeded 18S rRNA-tau fibril and **B)** AD PHF fold (PDB 5o3l). Protofilament interface contacts are shown for PHF (teal) and SF (light blue). G333 to E338 is buried in the protofilament interface of PHFs but would be accessible in straight filaments.

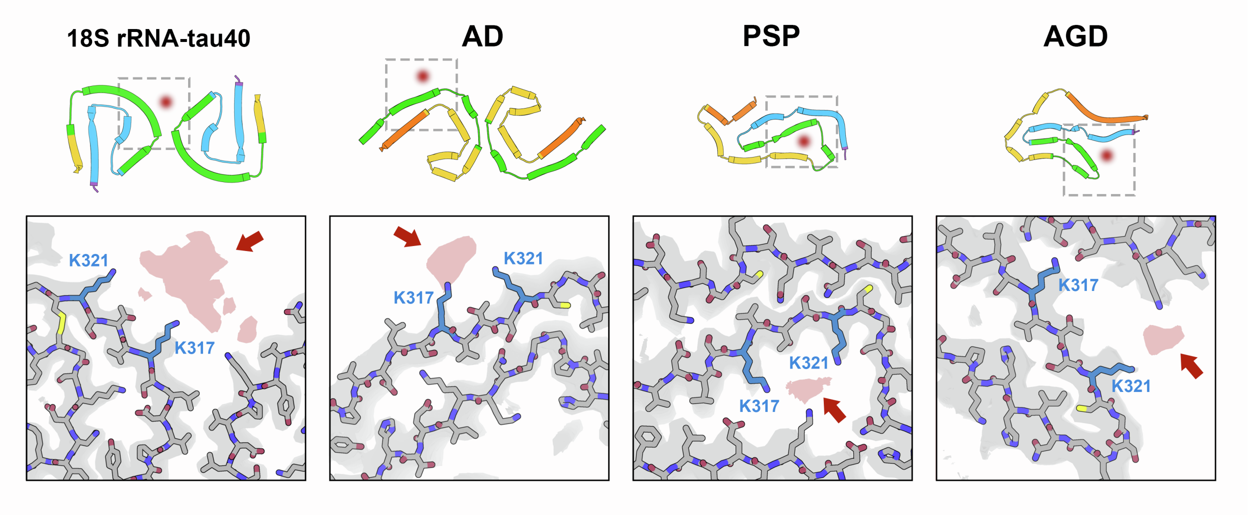

Figure S10. Interactions of K317 and K321 with residual density in 18S rRNA-tau fibrils and *ex vivo* tau fibrils. Cartoons of amyloid cores of unseeded 18S rRNA-tau fibril (left), AD PHF (middle left), progressive supranuclear palsy (PSP, middle right) and argyrophilic grain disease (AGD, right) tau folds. Dotted boxes indicate zoomed views, shown below, to highlight tau interaction with residual density (red). Cryo-EM maps and atomic models of unseeded 18S rRNA-tau fibril, AD PHF fold (PDB 5o3l, EMD 3741), PSP fold (PDB 7p65, EMD 13218) and AGD fold (PDB 7p6d, EMD 13226). K317 and K321 residues (blue) contact residual densities (red) indicated by red arrow.
